# Regulatory Pathways of Endophytic Fungi in Promoting Flavonoid Biosynthesis in Medicinal Plants

**DOI:** 10.3390/microorganisms14102141

**Published:** 2026-09-23

**Authors:** Yuan Gao, Haonan Dong, Zesen Fang, Silin Liu, Yan Xu, Yuyang Guo, Yang Yang, Jianghan Luo, Lijun Yan

**Affiliations:** School of Pharmacy, Engineering Research Center of Natural Antineoplastic Drugs, Ministry of Education, Harbin University of Commerce, Harbin 150076, China; donghaonany025@s.hrbcu.edu.cn (H.D.); fangzeseny024@s.hrbcu.edu.cn (Z.F.); liusilin@s.hrbcu.edu.cn (S.L.); xuyany023@s.hrbcu.edu.cn (Y.X.); guoyuyangy023@s.hrbcu.edu.cn (Y.G.); yangyang@hrbcu.edu.cn (Y.Y.); 102690@hrbcu.edu.cn (J.L.); ylj@hrbcu.edu.cn (L.Y.)

**Keywords:** endophytic fungi, medicinal plants, flavonoids, phenylpropanoid pathway, multi-omics technologies

## Abstract

Endophytic fungi form complex symbiotic associations with medicinal plants via long-term coevolution. Such cross-kingdom interactions substantially modulate flavonoid biosynthesis in host plants. This review dissects the mechanisms through which endophytic fungi elevate flavonoid accumulation in medicinal plants. We first summarize symbiotic and growth-promoting relationships between endophytic fungi and medicinal plants, outlining four core synergistic functions: nutrient supply, phytohormone homeostasis, stress resistance enhancement, and secondary metabolism activation. We then elaborate on three primary regulatory patterns underlying endophyte-modulated flavonoid biosynthesis, including direct activation of plant flavonoid biosynthetic pathways, indirect regulation via stress mitigation and host metabolic reprogramming, and autonomous flavonoid production by endophytic fungi. Notably, most current evidence for autonomous flavonoid biosynthesis by endophytic fungi is derived from axenic cultures, whereas its occurrence and quantitative contribution within colonized plant tissues remain to be established. We further discuss state-of-the-art technologies for untangling plant–fungus symbiotic interactions, including multi-omics integration, spatial metabolomics, single-cell sequencing, synthetic microbial consortia, and CRISPR-Cas genome editing. Lastly, we propose future research perspectives covering green cultivation of medicinal plants, targeted improvement of medicinal quality, and efficient microbial biosynthesis of flavonoids.

## 1. Introduction

Flavonoids are among the most important secondary metabolites in medicinal plants and contribute substantially to their pharmacological value. These compounds participate in plant defense, stress adaptation, pigmentation, and signaling, and many flavonoids exhibit antioxidant, anti-inflammatory, antimicrobial, antitumor, hepatoprotective, and cardioprotective activities [1]. Compared with other classes of plant secondary metabolites, such as alkaloids, terpenoids, and phenolic acids, flavonoids deserve particular attention because they are widely distributed across medicinal plant taxa, are closely associated with both plant ecological fitness and therapeutic efficacy, and their biosynthetic pathways are relatively well characterized [2]. Therefore, understanding how flavonoid biosynthesis and accumulation are regulated is important for improving the quality, efficacy, and sustainable production of medicinal plants.

The relationship formed between medicinal plants and endophytic fungi represents a highly synergistic form of biological interaction in nature. It profoundly regulates host plant growth, health, and stress adaptation, as well as the biosynthesis and accumulation of core bioactive compounds. Through long-term coevolution with medicinal plants, they have established a widespread and complex symbiotic relationship, which has been vividly described as a “secret wedlock” [3]. Endophytic fungi establish intimate and often mutually beneficial associations with their host plants, influencing host growth, stress adaptation, and specialized metabolism. The biosynthesis of flavonoids is controlled by plant genetic background, developmental stage, tissue type, and environmental conditions [4]. It is also strongly influenced by plant-associated microorganisms, especially endophytic fungi. This symbiotic relationship extends far beyond a simple host–symbiont association; it constitutes a functional unit characterized by a dynamic and ongoing dialogue of chemical signals. This interaction profoundly influences the host’s physiological ecology and stress resistance, particularly the synthesis and accumulation of bioactive secondary metabolites. It serves as a crucial bridge linking the plant’s genotype to its medicinal phenotype [5].

A growing body of evidence indicates that endophytic fungi can regulate flavonoid metabolism in medicinal plants through several interconnected mechanisms. Some endophytic fungi promote host growth and nutrient acquisition by producing phytohormones, solubilizing phosphorus, fixing nitrogen, or improving tolerance to drought, salinity, and other abiotic stresses [6]. In terms of enhancing disease resistance, they can protect the host by competing with pathogens for ecological niches and nutrients, producing antimicrobial metabolites, or systemically activating the plant’s immune defense system [7,8]. Others enhance plant defense by competing with pathogens, producing antimicrobial compounds, secreting fungal enzymes or siderophores, and activating host immune responses [9,10,11]. These physiological changes are often accompanied by shifts in secondary metabolism. In particular, fungal-derived elicitors and signaling molecules can activate plant signal transduction pathways, alter transcriptional regulation, and upregulate key genes involved in flavonoid biosynthesis, including genes encoding enzymes in the phenylpropanoid and flavonoid pathways [12,13]. Through these processes, endophytic fungi may increase the accumulation of specific flavonoids and thereby improve the medicinal quality of host plants.

Research on endophyte-mediated regulation of flavonoid biosynthesis has moved from simple isolation, culture, and metabolite screening toward integrated mechanistic analysis. Metagenomics and amplicon sequencing help characterize endophytic fungal communities associated with medicinal plants. Transcriptomics and proteomics reveal changes in host gene expression and protein abundance after fungal colonization or elicitation. Metabolomics further identifies changes in flavonoid profiles and related metabolic pathways. When combined, these approaches provide a systems-level understanding of how endophytic fungi influence flavonoid biosynthesis, accumulation, and transport in medicinal plants [14]. Spatial metabolomics also offers new opportunities to visualize the distribution of flavonoids and other metabolites at the plant–fungus interface, helping to clarify where and how metabolic interactions occur in host tissues [15]. Current studies on endophyte-modulated flavonoid metabolism span multiple levels of evidence, ranging from correlative phenotypic and omics associations, pathway-level signaling responses, functional regulatory events, and metabolic flux redistribution to definitive causal validation.

Therefore, the interaction between endophytic fungi and medicinal plants should be considered an important regulatory factor in flavonoid biosynthesis rather than merely a general symbiotic phenomenon. Clarifying the mechanisms by which endophytic fungi modulate flavonoid metabolism can provide theoretical support for improving medicinal plant quality and practical guidance for developing microbial fertilizers, biostimulants, biocontrol agents, and sustainable cultivation strategies [16]. Many existing reviews have independently summarized endophyte-medicinal plant symbiotic relationships, fungal elicitor-triggered plant secondary metabolism, flavonoid biosynthetic pathways, and metabolic alterations induced by mycorrhizal symbiosis. Nevertheless, these publications tend to focus on individual facets of plant–microbe interactions and rarely provide an integrated synthesis covering host-directed metabolic responses, stress-associated metabolic reprogramming, and fungal-derived metabolite production within holobiont systems. Accordingly, this review aims to synthesize current scattered research findings across these interrelated dimensions. We also seek to distinguish varying degrees of experimental support among published studies and summarize unresolved challenges to guide future mechanistic and translational investigations.

A literature search was conducted in PubMed, Web of Science, and Scopus, with the final search completed in May 2026. Keywords included “endophytic fungi,” “medicinal plants,” “flavonoids,” “phenylpropanoid,” “biosynthesis,” and “regulation,” used alone or in combination according to the syntax of each database. The search mainly covered publications from 2010 to 2026, with selected earlier landmark studies included for historical context. Peer-reviewed original articles and review papers were considered if they addressed endophyte-associated flavonoid production, metabolic regulation, host–microbe interactions, or mechanistic aspects in medicinal plants. Conference abstracts, dissertations, duplicate records, and studies with limited relevance were excluded.

## 2. Symbiosis and Growth Promotion Between Endophytic Fungi and Medicinal Plants

### 2.1. The Symbiotic Relationship Between Endophytic Fungi and Medicinal Plants

Endophytic fungi colonize medicinal plant tissues via natural openings, including stomata, lateral root fissures, and wound sites. Plasma membrane receptors of medicinal plants recognize fungal chitin fragments and oligosaccharide elicitors, triggering transient ROS bursts and cytoplasmic Ca^2+^ influx to activate basal immune responses. Distinct from pathogenic infection, compatible endophytic fungi suppress host PAMP-triggered immunity (PTI) and induce the expression of symbiosis-related genes such as CASTOR and POLLUX [17,18]. It should be noted that these molecular events are not broadly conserved across all non-mycorrhizal medicinal-plant endophytic fungi. In these model symbiosis systems, immune attenuation restricts fungal hyphae to epidermal and cortical intercellular spaces and prevents massive invasion into vascular bundles [19,20,21]. Critically, tissue colonization merely reflects endophytic residence rather than obligate mutualism: the interaction status can shift among neutral, growth-promoting, or weakly pathogenic modes, modulated by host genotype, fungal strain, and environmental context.

Endophytic fungi may be acquired horizontally from soil, air, or neighboring plants, but some fungi are seed-borne and can be transmitted vertically from parent plants to their offspring. Vertical transmission may facilitate the stable inheritance and early establishment of plant–fungus associations and may be particularly important in perennial medicinal plants or plants exposed to recurrent environmental stress. However, vertically transmitted fungi are not necessarily beneficial under all conditions. Their effects on host growth, defense, and flavonoid metabolism may remain dependent on host genetic background, tissue specificity, fungal abundance, plant developmental stage, and environmental context.

Endophytic fungi commonly colonize the internal root tissues of host plants as dark septate endophyte (DSE) hyphae, while adopting the characteristic orchid mycorrhizal pattern in members of the Orchidaceae [22,23]. Within leaves, the fungus primarily produces hyaline vegetative hyphae that grow along cell walls and occasionally generates chlamydospores. In addition, plant leaves also harbor diverse endophytic fungal communities [24]. The colonizing population of endophytic fungi is generally much smaller than the volume of plant tissue, but it can systematically influence host gene expression. For instance, during the early colonization stage, the endophytic fungus *Metarhizium anisopliae* establishes a symbiotic association with its host by activating key genes involved in the indole-3-acetic acid (IAA) biosynthetic pathway, thereby exerting systemic effects on host physiological processes [25], for example, promoting the synthesis of flavonoids.

Chemical signal dialogue governs host–fungus compatibility and colonization intensity in medicinal plant systems. Endophytic fungi secrete small signaling metabolites, while medicinal plants reciprocally release flavonoids, strigolactones, and jasmonic acid as feedback signals. As a typical example, *Serendipita indica* rearranges intracellular auxin transport and distribution to rebalance endogenous hormone crosstalk within host cells [26]. Such chemical communication collectively enhances plant tolerance to drought, salinity, and heavy metal stress. Stress mitigation reduces unnecessary metabolic expenditure on basal antioxidant defense and redirects metabolic flux toward the synthesis of pharmacologically valuable flavonoid compounds. The co-evolved “low invasiveness, intensive signal crosstalk” trait of medicinal plant–endophyte symbiosis lays a solid physiological foundation for fungal-mediated flavonoid biosynthesis regulation [27,28,29].

### 2.2. The Promotive Relationship Between Endophytic Fungi and Medicinal Plants

Growth-promoting symbiosis represents the most application-valued type of plant–endophyte interaction. Endophytic fungi can distinctly improve the biomass, root activity, accumulation of bioactive compounds, and cultivation adaptability of medicinal plants. The relevant mechanisms mainly involve nutrient modulation, phytohormone regulation, stress resistance enhancement, and secondary metabolism induction (Figure 1). Such growth-promoting effects have been widely verified in tissue culture, pot experiments and field trials. Common research methods include multi-omics technologies (transcriptomics, metabolomics, 16S and ITS sequencing) and isotope tracing analysis.

#### 2.2.1. Nutrient Acquisition and Uptake

Endophytic fungi secrete oxalic acid, citric acid, and phosphatase, converting insoluble soil phosphorus and potassium into bioavailable forms to optimize host mineral supply [30,31]. Representative strains include *Aspergillus niger*, *Penicillium* spp., and *Trichoderma* spp., which improve root architecture and boost nutrient absorption capacity, while *Piriformospora indica* facilitates uptake of nitrogen, phosphorus, and potassium to strengthen host biomass accumulation [32]. *Phomopsis liquidambaris* B3 elevates root IAA and ABA levels, raises soil ammonium nitrogen, and enhances plant nitrogen storage [33]. Endophytes also mobilize insoluble iron to support host physiological activity [34,35]. Improved mineral nutrition lays a material foundation for the enhanced carbon flux toward phenylpropanoid pathways, which is a prerequisite for elevated flavonoid biosynthesis in medicinal plants.

#### 2.2.2. Hormonal Growth Regulation

Endophytic fungi synthesize plant hormones such as IAA, gibberellins (GAs), zeatin riboside (ZR), and ABA, which directly promote cell division and elongation [36,37,38]. Endophytes isolated from *Oxalis corniculata*, such as *Aspergillus fumigatus* TS1 and *Fusarium proliferatum* BRL1, produce abundant IAA and gibberellins [39]. Mycorrhizal symbiosis reshapes the endogenous hormonal network via nutrient exchange, mediating ABA, auxin, SA, and JA signal cascades to modify antioxidant capacity and nutrient transport [40,41].

Cytokinins and auxins coordinately govern fungal colonization; SA and JA jointly balance symbiosis and stress responses to reinforce host adaptability [42]. Specific fungal metabolites (e.g., sterols secreted by *Ceratobasidium* GS2) regulate seed germination and organ development of medicinal orchids [43]. Crucially, these fungal-derived hormonal signals act as upstream regulatory switches to activate transcription factors that drive flavonoid structural gene expression in medicinal plants.

#### 2.2.3. Stress Resistance and Defense Activation

As natural elicitors, endophytic fungi release cell wall β-glucans and chitin oligosaccharides that trigger SA/JA-dependent systemic resistance and reduce pathogen lesions [44,45]. Meanwhile, endophytic fungi colonization significantly enhances host antioxidant enzyme activities and promotes the accumulation of non-enzymatic antioxidants, thereby alleviating oxidative damage under drought, salinity, high temperature, and heavy metal stress [46]. As vital symbiotic regulators, endophytic fungi sustain regular photosynthesis, nutrient absorption, and robust growth of medicinal plants under drought, salinity, heat, and heavy metal stress through coordinated osmoregulation, ion balance, and phytohormone modulation. Stable physiological homeostasis provides sufficient carbon skeletons to support continuous flavonoid biosynthesis [47,48,49]. Importantly, stress-induced antioxidant responses triggered by endophytic fungi substantially reshape host metabolic flux toward the phenylpropanoid pathway, which is the core upstream route for flavonoid biosynthesis.

Endophytic fungi secrete metabolites, such as extracellular polymeric substances (EPS), which further function as non-contact biostimulants. EPS significantly increases the activity of phenylalanine ammonia-lyase (PAL), the rate-limiting enzyme of flavonoid synthesis, thereby directly promoting flavonoid accumulation in medicinal plants [50,51]. Overall, endophyte-activated stress defense responses do not merely improve plant stress adaptability. Such defense priming serves as a key upstream signal that reprograms secondary metabolism and substantially promotes flavonoid overaccumulation in medicinal plants under both normal and stressful conditions.

#### 2.2.4. Secondary Metabolism and Quality Improvement

Endophytic fungal colonization reshapes host secondary metabolism, with flavonoid accumulation serving as the most representative functional phenotype. A large number of medicinal plant cases verify that endophyte inoculation substantially elevates total flavonoid contents; for instance, *Trichoderma atroviride* D16 significantly increases phenolic and flavonoid yields in *Salvia miltiorrhiza* [52].

Beyond inducing plant endogenous flavonoid synthesis, endophytic fungi themselves produce flavonoid derivatives as bioactive elicitors. Fungal acetylated flavonoids synergize with host metabolites to enhance overall antioxidant and medicinal activity, forming a dual flavonoid source within the symbiotic system [53]. Collectively, the combined effects of nutrient supply, hormonal modulation, and stress priming mediated by endophytic fungi converge to drive flavonoid enrichment in medicinal plants. Such microbial induction strategies lower chemical fertilizer and pesticide reliance, supporting eco-friendly cultivation and consistent medicinal material quality (Table 1).

## 3. Major Flavonoid Compounds in Medicinal Plants and Their Effects

Flavonoids constitute an important class of secondary metabolites widely distributed in medicinal plants and are characterized by a common C6–C3–C6 skeleton [69]. Based on the oxidation state of their central C6–C3–C6 skeletons and variations in their substitution patterns, flavonoids are generally categorized into subclasses such as flavanols, flavanones, flavones, isoflavones, and biflavones [70,71]. In medicinal plants, flavonoids commonly occur as glycosides rather than exclusively as free aglycones. Glycosylation involves the conjugation of a sugar moiety to the flavonoid skeleton and can substantially affect compound solubility, stability, cellular transport, tissue distribution, metabolism, and biological activity.

Different plant species accumulate distinct flavonoid constituents with varied proportions, thereby endowing them with diverse and complementary pharmacological profiles [72]. Among common medicinal plants, different types of flavonoids each play a key role [73]. For example, quercetin and kaempferol, common flavonoid compounds, occur widely in medicinal plants and, along with rutin, exert strong antioxidant effects [74]. Flavanols represented by epicatechin constitute the principal components of tea polyphenols. Accumulating evidence demonstrates that they exert multiple health-beneficial bioactivities, including antioxidant, anticancer, cardioprotective, antimicrobial, antiviral, and neuroprotective effects [75]. Flavonoids (e.g., apigenin) and flavanones (e.g., naringin) also confer prominent anti-inflammatory and vasoprotective effects in multiple medicinal plants [76,77]. Particularly noteworthy are the bioactive constituents of ginkgo leaves, which are abundant in biflavonoids including ginkgolides. Owing to their multi-target regulatory effects on the nervous system, these compounds exhibit prominent potential in the intervention of neurodegenerative disorders such as Alzheimer’s disease [78,79]. Another prominent subclass is flavonolignans, represented by silybin from milk thistle, which are formed via the condensation of a flavonoid and a phenylpropanoid unit and exhibit hepatoprotective effects [80].

The molecular mechanisms responsible for the bioactivity of these flavonoids have been increasingly elucidated. A core pathway is the activation of the intracellular NRF2 signaling cascade, which serves as a master regulator of cellular antioxidant responses. This cascade triggers the expression of multiple detoxification enzymes and antioxidant proteins, enabling cells to combat oxidative stress-induced damage [81]. Furthermore, these flavonoids can effectively suppress several core pro-inflammatory signaling pathways, such as TLR and NF-κB. They also downregulate the activity of inflammatory mediators including cyclooxygenase-2 (COX-2), thus exerting potent broad-spectrum anti-inflammatory effects [82]. In the cardiovascular system, specific flavonoids are able to dilate coronary arteries, elevate coronary blood flow, and reduce vascular fragility. This confers a solid theoretical basis for their application in the prevention and treatment of hypertension, coronary heart disease, and angina pectoris [83]. Furthermore, the antibacterial and antiviral activities of these flavonoids are closely associated with their capacity to disrupt microbial life activities [84]. It should be noted that many of the reported pharmacological effects have been demonstrated using purified compounds, flavonoid-enriched fractions, or crude plant extracts rather than plant materials whose flavonoid profiles were specifically modulated by endophytic fungi.

## 4. Effects of Endophytic Fungi on Host Plants and Flavonoid Synthesis Regulation

Plants and endophytic fungi have formed intricate interaction networks via long-term coevolution [85]. Flavonoid accumulation in medicinal plants is regulated by multiple host and environmental factors, and endophytic fungi may contribute to this process through several direct or indirect routes. High-throughput sequencing has been used to characterize the endophytic fungal diversity of tartary buckwheat, together with the screening of fungal strains that may improve flavonoid accumulation and plant growth. In one study, the relative abundances of fungal genera, including *Bipolaris*, *Hymenula*, and *Colletotrichum*, were positively correlated with the concentrations of major flavonoids, such as rutin and quercetin [86]. These findings suggest a potential ecological or functional association between the fungal community and flavonoid accumulation; however, they do not demonstrate that these fungal genera directly regulate flavonoid biosynthesis.

The observed correlations may also reflect the effects of host genotype, tissue chemistry, plant developmental stage, or environmental conditions on both fungal community composition and flavonoid accumulation. Conversely, flavonoids or other host metabolites may influence fungal recruitment, colonization, or relative abundance. Therefore, the reported relationships should be regarded as correlative evidence rather than experimentally demonstrated causality. Direct regulatory effects would require additional evidence, such as controlled inoculation experiments, fungal strain-specific colonization analysis, temporal measurements of fungal abundance and host responses, fungal mutants, purified fungal elicitors, host gene perturbation, or isotope-tracing and metabolic-flux analyses.

On this basis, the potential interaction between endophytic fungi and flavonoid metabolism is better represented as an interconnected network involving possible host-pathway responses, fungal-derived metabolites, and changes in the host growth or stress environment (Figure 2). These processes may overlap, but their relative contributions and causal relationships remain to be established in most medicinal plant systems.

### 4.1. Biological Induction and the Activation of Plant Metabolic Pathways

This is the most fundamental and widespread mechanism. As efficient bioinducers, endophytic fungi trigger mild biological stress in host plants. This activates host defense signaling networks and further induces secondary metabolic pathways such as flavonoid biosynthesis. Figure 3 illustrates the signal transduction cascade from fungal elicitors to flavonoid biosynthesis genes. JA and SA signaling pathways also contribute greatly to this process [87]. For instance, liquorice (*Glycyrrhiza glabra* L.) is a flavonoid-rich medicinal plant. Its flavonoid synthesis is modulated by jasmonic acid-related hormones. The differential regulatory effects of methyl jasmonate (MeJA) and salicylhydroxamic acid (SHAM) on flavonoid biosynthesis are as follows: MeJA promotes flavonoid synthesis by activating the JA signaling pathway and downstream MYB/bHLH transcription factors, while SHAM inhibits this pathway [88]. In more specific symbiotic relationships, such as the mutualistic symbiosis between arbuscular mycorrhizal fungi (AMF) and host plants, these fungi can trigger extensive metabolic reprogramming in host cells. In this way, AMF improve the medicinal properties of Polygala tenuifolia and boost the accumulation of flavonoids and other metabolites [89]. Hormonal signals also exhibit obvious crosstalk. For example, increased jasmonate levels can simultaneously boost ginsenoside and flavonoid synthesis in cambial meristem cells of *Panax quinquefolius* [90]. Mycorrhizal fungi provide an important comparative context for understanding fungal effects on flavonoid metabolism but should be distinguished from non-mycorrhizal endophytic fungi.

At the molecular level, this activation is even more precise. When *Botryosphaeria dothidea* J46 infects drought-stressed buckwheat, it specifically upregulates genes encoding key enzymes in the host phenylpropanoid biosynthesis pathway, including cinnamic acid 4-hydroxylase (C4H), chalcone synthase (CHS), and chalcone isomerase (CHI). This process further enhances flavonoid biosynthesis to help the host cope with drought stress. By contrast, *Irpex lacteus* J79 indirectly facilitates flavonoid synthesis by elevating the expression of photosynthetic genes, which increases energy supply and the accumulation of L-phenylalanine precursors. These two strains act synergistically to strengthen host drought tolerance through distinct regulatory pathways [91].

One example illustrates how fungal secreted proteins may mediate fungus–host protein interactions. The fungal PgSCP protein interacts with a citrus transcription factor and activates PR1-like defense genes [92]. This finding provides a plausible analogy for signal transduction during endophyte-host crosstalk. However, this study does not offer direct experimental evidence connecting PgSCP to the expression of flavonoid biosynthetic genes or changes in flavonoid metabolite levels. This specific direct interaction reveals a novel mechanism by which fungi precisely modulate plant defense-related metabolic networks, particularly flavonoid biosynthesis.

### 4.2. Environmental Stress Buffering and Subsequent Metabolic Reprogramming

Fungi also exert profound indirect impacts on host metabolic pathways and metabolic fluxes. They strengthen host stress resistance, which is referred to as the environmental buffering effect. Flavonoids exert bioactive functions to inhibit pathogens and alleviate plant disease symptoms [93]. Flavonoids have also been shown to correlate positively with the antioxidant activity of plants [94]. Endophytic fungi reshape plant microbial communities and inhibit the colonization of pathogenic fungi. This boosts plant stress tolerance and rebalances resource allocation between primary and secondary metabolism [47,95]. The root endophytic fungus *Piriformospora indica* can activate the phenylpropanoid pathway. It further promotes the synthesis of flavonoids and flavanols. This improves soybean growth, nutrient absorption, and abiotic stress tolerance [96]. The effect of endophytic fungi on flavonoid production is context-dependent and governed by growth–defense trade-offs. When endophytic fungi trigger mild stress responses, phenylpropanoid and flavonoid biosynthesis can be enhanced for antioxidant and defensive purposes. In other scenarios, endophytic fungi alleviate host oxidative stress. With reduced antioxidant demand, the host may decrease flavonoid synthesis and reallocate carbon resources to support vegetative growth. The outcome of the growth–defense trade-off is also conditioned by carbon-source availability: when photosynthates and soluble sugars are sufficient, plants may sustain both growth and flavonoid biosynthesis, whereas under carbon limitation, the diversion of carbon skeletons toward defense-related specialized metabolism may impose a more pronounced growth cost. The final outcome depends on several interacting variables: stress intensity, carbon availability, plant developmental stage, and the lifestyle of the colonizing fungus.

Under drought stress, beneficial fungi relieve plant physiological damage. They induce proline accumulation, increase SOD and POD activities, and maintain root development and photosynthetic performance [97]. Endophytic fungi function as reprogrammers of plant metabolic networks. They do not regulate only a single pathway but instead comprehensively modulate the flux of multiple pathways, including flavonoid metabolism. Similarly, AMF reprogram root metabolism in *Polygala tenuifolia* through pathways of unsaturated fatty acids, diterpenes, and flavonoids [89]. After fungi mitigate plant oxidative stress, plants reduce the massive synthesis of antioxidant flavonoids. This further shifts the overall metabolic priority of the host. This process reflects resource competition. *Paraphoma chrysanthemicola* affects carbohydrate metabolism and suppresses SA signaling in *Codonopsis pilosula*, thus reshaping the trade-off between primary and secondary metabolism [98]. This metabolic trade-off can be seen in *Aspergillus terreus* during the synthesis of physcion, a derivative of emodin. A balance exists between biosynthetic efficiency and cellular adaptability. Loss of the detoxification-related P450 enzyme raises physcion yield. However, this change weakens the robustness of the fungal strain [99].

After the fungus alleviates oxidative stress, the plant reduces the overproduction of antioxidant flavonoids, thereby redirecting metabolic resources toward growth, reflecting a resource trade-off between stress resistance and growth. Alternatively, endophytic fungi help plants acquire sufficient resources. This allows plants to produce more diverse defensive compounds, including flavonoids. Endophytic fungi can produce abundant defensive bioactive compounds, especially antioxidants. The endophytic strain *Phoma* sp. SK5 was isolated from African clubmoss. Its metabolites scavenge multiple free radicals such as DPPH, ABTS, and H_2_O_2_. This strain exhibits strong antioxidant capacity [100]. This demonstrates that, within a symbiotic system, these capabilities of fungi can bolster the plant’s overall defense mechanisms, enabling it to cope with a wider range of stresses. This buffering effect keeps flavonoid synthesis stable and plastic in plant–fungus symbiosis under varied environments.

### 4.3. Intrinsic Contribution of Endophytic Fungi to Flavonoid Biosynthesis

Traditional studies view flavonoids as typical plant metabolites. However, recent groundbreaking research shows that some fungi can independently synthesize flavonoids via novel pathways. This finding has greatly updated our understanding of flavonoid origin and evolution. Notably, these fungi have evolved a unique flavonoid biosynthetic enzyme system, which is completely different from that of plants.

The most significant discovery comes from the plant-associated fungus *Pestalotiopsis fici*. Researchers identified an NRPS-PKS hybrid enzyme named FnsA in this fungus. This enzyme uses free p-coumaric acid or p-hydroxybenzoic acid as direct substrates. It catalyzes the synthesis of naringenin, a key flavonoid precursor [101]. This pathway completely skips two key steps in plant flavonoid synthesis. It avoids the formation of coumaroyl-CoA (the essential substrate activation) and the CHS-catalyzed condensation reaction. Thus, it represents a brand-new flavonoid biosynthetic pathway. This not only represents a significant addition to our understanding of the flavonoid biosynthetic pathway but also demonstrates the convergent evolution and diversity of metabolic pathways in nature.

Further research has confirmed that this is not an isolated case. In *Aspergillus luminescens*, a complete gene cluster responsible for the synthesis of chloroflavonoids has been identified. This cluster comprises CfoA (an NRPS-PKS hybrid enzyme responsible for constructing the chalcone skeleton), CfoK (a chalcone isomerase with a novel catalytic mechanism), and CfoJ (a novel flavonoid synthase) [102]. These fungal enzymes show obvious differences from plant homologues in sequence and catalysis. They build a full plant-independent pathway for flavonoid biosynthesis. At present, virtually all microbial cell factories for flavonoids have been constructed by heterologously engineering plant pathways. These findings possess important theoretical significance. They allow the novel enzymes to be applied in microbial cell factories, enhancing the efficient synthesis of high-value flavonoids and offering promising technical prospects.

In summary, endophytic fungi regulate plant flavonoid biosynthesis through a synergistic network with multiple levels and mechanisms. Some strains belonging to *Colletotrichum* and *Bipolaris* illustrate the functional plasticity of endophytic fungi. Certain strains within these genera can behave as pathogens under specific conditions, while other strains can synthesize flavonoids autonomously or trigger host flavonoid accumulation. It is important to note that these traits are strain-specific and context-dependent [86]. Evidence at the genus level cannot support the claim that a single strain possesses all of these functions simultaneously. Activation, endogenous synthesis, and environmental buffering are closely intertwined in symbiosis rather than functioning separately. They sustain dynamic equilibrium and collectively determine the flavonoid metabolism of host plants. Table 2 summarizes the pathways by which some endophytic fungi promote flavonoid biosynthesis in their host plants.

Most supporting evidence for this biosynthetic capacity originates from axenic fungal culture experiments. It is critical to distinguish three separate scenarios: flavonoid production by fungi in pure culture, flavonoid biosynthesis by fungi while colonizing host plant tissues, and a quantitatively substantial contribution of fungal flavonoids to the total flavonoid pool within medicinal plants. Evidence obtained from axenic culture confirms the inherent biosynthetic potential of these fungi, but it cannot prove that flavonoid synthesis occurs during endophytic colonization, nor can it demonstrate that fungal-derived flavonoids make up a meaningful fraction of the total flavonoids detected in host plants. While these BGCs are promising for developing microbial cell factories, direct in planta evidence is still needed to evaluate the fungal contribution to plant flavonoid pools under symbiotic conditions.

## 5. New Techniques for Studying the Interactions Between Endophytic Fungi and Medicinal Plants

Research on the interactions between endophytic fungi and medicinal plants has emerged as a research hotspot in biotechnology and pharmaceutical science. Current studies mainly focus on the bioactive metabolites produced by endophytic fungi in host plants, as well as their growth-promoting effects on medicinal plants [6]. In recent years, benefiting from the rapid advances in high-throughput sequencing, high-resolution imaging, artificial intelligence, and computational biology, mechanistic research on interactions between medicinal plants and endophytic fungi has moved beyond conventional isolation, cultivation, and chemical analysis, stepping into a new era of multi-dimensional and systematic analysis [118]. This field is undergoing a fundamental paradigm shift. Its research focus has evolved from traditional isolation and identification to multi-dimensional, cross-scale, and engineering-oriented exploration. Emerging technologies in integrated applications have deepened our insight into this intricate symbiotic system. They also bridge fundamental research and industrial translation efficiently [119]. These advanced technologies uncover the intricate molecular interactions behind symbiosis. Meanwhile, they provide powerful tools to precisely regulate the biosynthesis of bioactive components in medicinal plants and to explore innovative microbial resources (Figure 4).

### 5.1. Multi-Omics Integration

Multi-omics integration enables systematic and quantitative dissection of the core regulatory mechanisms underlying plant–fungal symbiosis, including biological induction, environmental metabolic buffering, and fungal endogenous flavonoid biosynthesis. Traditional endophyte research mostly focuses on fungal isolation, culture and metabolite bioactivity screening. This method, however, cannot reflect the dynamic interaction mechanisms between host and endophyte. Multi-omics integration now stands as the core tool to unpack this interaction network [14]. By integrating multi-dimensional data from metagenomics, transcriptomics and metabolomics, this study systematically reveals how endophytic fungi influence gene expression, metabolite synthesis, and stress responses in medicinal plants. For example, a study focusing on *Atractylodes lancea* identified *Paraphoma* sp. and *Paraburkholderia aromaticivorans* as the endophytic fungi and bacteria with the most potent growth-promoting effects. Using multi-omics techniques combined with phenotypic analysis, the study systematically elucidated the distinct regulatory mechanisms by which these two organisms modulate *Atractylodes lancea* [120].

Multi-omics profiling can uncover correlations between fungal colonization, host transcriptional changes, and flavonoid accumulation. However, correlative multi-omics data alone cannot confirm causal regulatory mechanisms. Causal validation requires targeted functional experiments, such as construction of fungal mutants, host gene knockouts or knockdowns, genetic complementation assays, treatment with purified fungal elicitors, isotope-labelled precursor tracing, axenic or gnotobiotic culture systems, and time-resolved temporal experiments. These approaches help separate coincidental association from true regulatory control.

Advances in spatial metabolomics have enabled intact-tissue analysis. This technique directly visualizes the spatial distribution and translocation of metabolites between medicinal plant tissues (roots, stems, leaves) and their endophytic fungi. It offers an unprecedented perspective for deciphering metabolic crosstalk within host–microbe symbiosis [121,122]. Spatial metabolomics still faces a key bottleneck: spatial resolution and detection sensitivity are hard to balance. High spatial resolution usually comes with reduced metabolome coverage, and the technique is limited in the in situ identification of trace fungal metabolites. Furthermore, the time lag between metabolite production and detection may lead to the loss of dynamic information. In the future, isotope labeling or live-cell imaging can be integrated to achieve real-time tracking. Endophytic fungi do not exert uniform effects on the host’s entire metabolic system. Instead, they induce distinct metabolic profiles in different organs (roots, stems, leaves) or distinct regions of the same organ (e.g., the head, main body, and tail of Angelica sinensis root) via specific signaling molecules [123]. Spatial metabolomics plays a pivotal role in exploring plant–microbe interactions by characterizing the spatial specificity of metabolism and microbiome across medicinal plant parts.

High-throughput sequencing combined with multi-omics integrated analysis has become the core approach to dissect plant–microbe interaction mechanisms [14]. Metagenomics bypasses microbial culture and directly sequences total fungal DNA in plant tissues. It comprehensively characterizes the species composition and functional gene pool of endophytic fungal communities [124]. However, it suffers from limited species resolution, uncertain functional gene annotation, and insufficient spatiotemporal dynamic information. Based on this, the integrated application of transcriptomics and metabolomics, namely, transcriptome–metabolome correlation analysis, can directly link gene expression changes in fungi and host plants with the accumulation dynamics of key active ingredients (e.g., polysaccharides, flavonoids, alkaloids) and precisely identify the key metabolic pathways and regulatory nodes of their interactions [125]. For example, combined comparative transcriptomic and metabolomic analyses have uncovered the molecular mechanisms by which the endophytic fungus *Pestalotiopsis* sp. DO14 promotes the accumulation of polysaccharides and flavonoids in *Dendrobium officinale* [126]. Furthermore, proteomics and protein–protein interactomics can identify effector proteins and signaling molecules that directly function at the interaction interface, providing direct evidence for deciphering the establishment of symbiosis [127].

### 5.2. Single-Cell and High-Resolution Imaging Technologies

Single-cell sequencing and high-resolution imaging effectively resolve cellular heterogeneity in fungal biological induction. These techniques capture cell-specific signal perception and flavonoid regulatory responses obscured in conventional bulk analyses. To observe the interaction process at a finer scale, breakthroughs have been made in single-cell and high-resolution imaging technologies. Single-cell RNA sequencing (scRNA-seq) distinguishes the specific transcriptional responses of different host plant cell types (e.g., epidermal cells, vascular bundle cells) to fungal colonization, thereby uncovering the spatial heterogeneity of plant–endophyte interactions [128,129,130]. Plant cells feature thick cell walls and compact intercellular connections. This makes protoplast isolation quite difficult. Meanwhile, fungal RNA only accounts for an extremely small proportion in plant cells, greatly challenging the sensitivity and accuracy of data interpretation. By contrast, advanced imaging approaches such as confocal microscopy allow non-invasive in vivo observation. They can visualize and quantify the three-dimensional distribution and colonization patterns of fungal hyphae inside plant tissues [131]. These techniques closely link molecular mechanisms to cellular and histological phenotypes. They enable full-scale observation, ranging from the macroscopic to the microscopic level.

### 5.3. Synthetic Biology and Gene Editing

Synthetic biology and CRISPR-based gene editing technologies provide targeted genetic tools to verify and optimize all three regulatory modes, especially enabling functional validation of silent fungal BGCs responsible for endogenous flavonoid synthesis and optimizing fungal inductive regulatory efficiency. At the genetic level, synthetic biology and gene editing serve as powerful tools. They support the targeted mining and modification of the metabolic potential of endophytic fungi. Biosynthetic gene cluster (BGC) editing based on the CRISPR-Cas system empowers researchers to precisely manipulate target gene clusters in endophytic fungi. These clusters govern the biosynthesis of specific bioactive compounds, including polyketides, terpenoids, and meroditerpenoids. Such precise operations include knockout, activation, and heterologous reconstruction, further elucidating biosynthetic pathways and generating novel derivatives.

As a typical example, in vitro CRISPR-Cas9 ribonucleoprotein editing was applied to delete the global negative regulator mcrA in Aspergillus wentii. This manipulation awakened multiple silent polyketide BGCs and boosted the biosynthesis of 17 secondary metabolites. Further dual knockout of the dominant PKS gene gedC eliminated abundant emodin derivatives, unmasking an undescribed metabolite designated aspergillus acid E alongside known aspergillus acid B [132]. This strategy not only accelerates the discovery of drug lead compounds but also enables the heterologous expression of high-value metabolites in engineered strains and the establishment of sustainable bioproduction platforms.

Fueled by large-scale datasets from these advances, bioinformatics and synthetic biology support the rational design and regulation of plant–endophyte interaction systems. Bioinformatics mining of metagenomic and transcriptomic data can predict cryptic BGCs in endophytic fungi, revealing their capacity to produce new natural products [133,134]. Against this backdrop, synthetic microbial communities (SynComs) technology has emerged as a powerful research frontier. SynComs further upgrades the regulatory system by artificially assembling functional endophytic consortia, realizing standardized optimization of fungal-induced flavonoid promotion and environmental buffering effects under controllable conditions. Artificial symbiotic systems can be constructed by assembling multiple endophytic fungi with specific functions, such as growth promotion and metabolic induction. When inoculated into medicinal plants, these synthetic communities can be systematically assessed for their capacity to improve the yield and quality of medicinal materials. This advances the research paradigm from describing natural symbiosis to rationally designing functional symbiosis [57,134,135].

Current SynComs designs mostly rely on simplified binary or ternary interactions. Hence, they can hardly recapitulate the complex competitive and cooperative relationships among multiple species in natural niches. Moreover, laboratory conditions differ greatly from field environments. Such discrepancies frequently lead to colonization failure or functional attenuation of synthetic microbial communities after transplantation. Future research should optimize the composition and stability of SynComs under conditions that better mimic natural ecological dynamics. In parallel, synthetic biology strategies can be used to engineer metabolic pathways in endophytic fungi, enabling the efficient production of high-value plant-derived drug precursors.

Emerging technologies such as multi-omics integration, single-cell analysis, high-resolution imaging, and synthetic biology have greatly advanced the study of interactions between endophytic fungi and medicinal plants. Collectively, these technical systems complement and verify one another: multi-omics clarifies molecular pathways, single-cell imaging resolves spatial and cellular heterogeneity, and gene editing achieves functional verification and industrial transformation of the three core regulatory mechanisms. These findings advance our mechanistic understanding of plant–endophyte symbiosis and foster the development of innovative strategies. They enable targeted quality improvement in medicinal plants through microbiome engineering and facilitate the sustainable discovery of novel endophytic fungal functions.

## 6. Challenges and Future Trends

### 6.1. Challenges

Current understanding of endophytic fungi-mediated flavonoid modulation in medicinal plants is largely built upon correlative molecular and physiological observations, yet major methodological and conceptual limitations continue to constrain mechanistic certainty and practical translation of existing findings. Prevailing studies heavily depend on multi-omics profiling, community association analysis, and phenotypic detection, which often capture statistical covariation rather than causal regulatory relationships [136]. Interpretations involving functional terms such as regulation, activation, and metabolic reprogramming are frequently generalized without rigorous genetic, biochemical, or functional validation. The absence of standardized verification systems—including fungal genetic modification, host gene perturbation, genetic complementation, purified elicitor treatment, isotopic tracing, and gnotobiotic cultivation—leads to widespread overinterpretation of associative data and weakens the reliability of current flavonoid regulatory models.

Functional plasticity inherent to plant–fungal symbiosis further complicates the predictable manipulation of flavonoid metabolism. Endophytic fungi reside along a dynamic endophyte–pathogen continuum, whereby beneficial metabolic phenotypes observed under controlled laboratory conditions are not constitutive or stable. Host genetic background, developmental status, soil nutrient availability, ambient stress intensity, temperature fluctuation, and fungal colonization density collectively reshape symbiotic outcomes. Excessive fungal proliferation within plant tissues can disrupt cellular homeostasis, trigger intense immune responses, and compromise both plant growth and secondary metabolism, resulting in loss of flavonoid-promoting effects or even pathogenic damage under field conditions [137]. Such context-dependent functional instability creates substantial uncertainty for strain screening and large-scale agricultural application.

In translational research, the evaluation system for medicinal plant quality remains oversimplified. Flavonoid concentration is commonly adopted as the sole indicator of improved medicinal value, ignoring critical trade-offs between secondary metabolite enrichment and overall plant productivity. Elevated tissue flavonoid concentration does not equate to higher total metabolite yield per plant, as fungal induction of secondary metabolism often suppresses vegetative biomass accumulation. Variations in metabolic profiles, pharmacological bioactivity, and batch-to-batch consistency further determine practical medicinal quality, which are rarely systematically assessed in current endophyte studies [138].

Advanced molecular technologies available in model microbial and plant systems remain underutilized in medicinal plant–endophyte research. Although single-cell resolution sequencing and CRISPR-based genome editing offer powerful tools for resolving cell-specific metabolic regulation and validating fungal biosynthetic gene clusters, most relevant applications remain conceptual and have not been empirically validated within native medicinal plant symbiotic systems [16].

### 6.2. Future Trends

Future research on endophyte-modulated flavonoid metabolism should prioritize the establishment of causal and mechanistically rigorous frameworks to replace correlative interpretation dominating the current literature. Systematic functional validation strategies, including targeted fungal mutagenesis, host gene knockdown, metabolite tracing, and sterile gnotobiotic culture systems, will enable clear differentiation between statistical association and genuine regulatory causality. Standardized grading of evidence strength, covering transcriptomic, biochemical, metabolomic, and direct microbial biosynthesis evidence, will improve the objectivity and reproducibility of mechanistic summaries in future synthetic studies [139].

Greater emphasis should be placed on resolving the context-dependent plasticity of endophytic fungal functions. Comprehensive assessment across variable host genotypes, developmental stages, environmental conditions, and colonization intensities will help define stable beneficial symbiotic thresholds. Explicit consideration of the endophyte–pathogen continuum will prevent overgeneralization of laboratory-derived beneficial phenotypes and improve the reliability of screened microbial resources for field application.

A multi-dimensional evaluation system is required for assessing endophyte-induced medicinal quality improvement. Future translational studies need to comprehensively integrate flavonoid concentration, total metabolic yield, plant biomass, secondary metabolite profiling, pharmacological activity, and production stability. Balanced evaluation of growth–defense trade-offs will support more scientific and practical assessments of endophyte inoculation effects [140].

A major future direction is the application of artificial intelligence and machine learning to decode complex multi-omics datasets. Transcriptomics, proteomics, metabolomics, epigenomics, microbiome profiling, and spatial metabolomics generate high-dimensional data that are difficult to interpret using conventional correlation-based methods alone. Machine learning models can be used to identify key regulatory nodes, predict gene–metabolite associations, infer transcription factor–target relationships, and prioritize candidate fungal elicitors or plant receptors for functional validation.

Network-based learning, graph neural networks, random forest models, Bayesian inference, and multi-omics integration algorithms may be particularly useful for reconstructing plant–fungus regulatory networks. In the future, AI-assisted models could help predict which endophytic fungi are most likely to enhance flavonoid biosynthesis in specific medicinal plants and under defined environmental conditions. However, these predictions must be supported by standardized datasets, well-annotated fungal genomes, reproducible colonization systems, and experimental validation [141].

Adaptation and application of cutting-edge molecular techniques will drive deeper mechanistic exploration in this field. Optimization of single-cell transcriptomics, targeted gene editing, and multi-omics integration will facilitate identification of cell-type-specific regulatory events and uncharacterized fungal functional BGCs. In planta verification of autonomous fungal flavonoid biosynthesis represents a core unresolved issue worthy of sustained research attention [16].

Advancing laboratory discoveries toward agricultural practice will become an essential developmental direction. Systematic field trials are needed to validate the stability, environmental adaptability, and safety of flavonoid-promoting endophytic strains. Establishment of standardized inoculant preparation, delivery, and field management protocols will ultimately promote the industrial utilization of beneficial endophytic resources for high-quality medicinal plant cultivation [140].

## 7. Conclusions

The association between endophytic fungi and medicinal plants is the result of long-term ecological adaptation and co-evolution. Rather than acting through a single regulatory route, endophytic fungi influence flavonoid biosynthesis through a multi-level network involving nutrient acquisition, phytohormone modulation, stress tolerance induction, defense signaling, and secondary metabolism activation.

These interactions can promote medicinal plant growth and enhance the accumulation of bioactive compounds both directly and indirectly. Endophytic fungi extensively synthesize stimulatory metabolites that affect the synthesis of compounds in host plants. The same fungal isolate may exert beneficial, neutral, or even adverse effects depending on the host species, plant developmental stage, tissue type, environmental condition, and colonization intensity. Therefore, future studies should move beyond descriptive observations of plant–fungus association and establish causal links among fungal colonization, host signaling, transcriptional regulation, enzyme activity, metabolic flux, and flavonoid accumulation. This requires integrated experimental systems combining gnotobiotic cultivation, isotope tracing, gene perturbation, metabolomics, and spatially resolved imaging. Such approaches will help distinguish whether flavonoid enhancement results from improved plant growth, elicitor-triggered defense responses, altered precursor supply, or direct fungal metabolic contribution.

Another important prospect is the rational design of synthetic microbial communities. Compared with single-strain inoculation, synthetic microbial communities may provide more stable and multifunctional benefits, including nutrient mobilization, stress resistance, disease suppression, and secondary metabolism enhancement. However, SynCom construction for medicinal plants still lacks standardized frameworks. Future SynCom development should include systematic procedures for strain isolation, genome sequencing, safety evaluation, functional screening, compatibility testing, community assembly, colonization monitoring, and ecological risk assessment. In addition, field validation should not rely only on plant biomass or metabolite content but should also include community stability, host specificity, persistence across growth stages, environmental adaptability, and potential effects on native microbiota. Standardized experimental designs, shared metadata, reproducible inoculation protocols, and multi-site field trials are essential for translating SynComs from controlled environments to ecological cultivation systems.

The deep integration of multi-omics, spatial metabolomics, AI-assisted prediction, synthetic biology, and SynCom engineering is driving a paradigm shift from the description of natural endophytic associations to the rational design of functional plant–microbe systems. In this emerging framework, endophytic fungi are not only ecological partners of medicinal plants but also biological resources for regulating plant specialized metabolism and constructing microbial platforms for flavonoid biosynthesis. Future research should clarify the molecular dialogue between fungal effectors and plant receptors, identify the regulatory nodes controlling flavonoid metabolic flux, and develop standardized engineering and field-validation strategies. Such advances will provide a stronger theoretical basis and technical support for the green cultivation, quality improvement, and sustainable production of bioactive ingredients in medicinal plants.

## Figures and Tables

**Figure 1:**
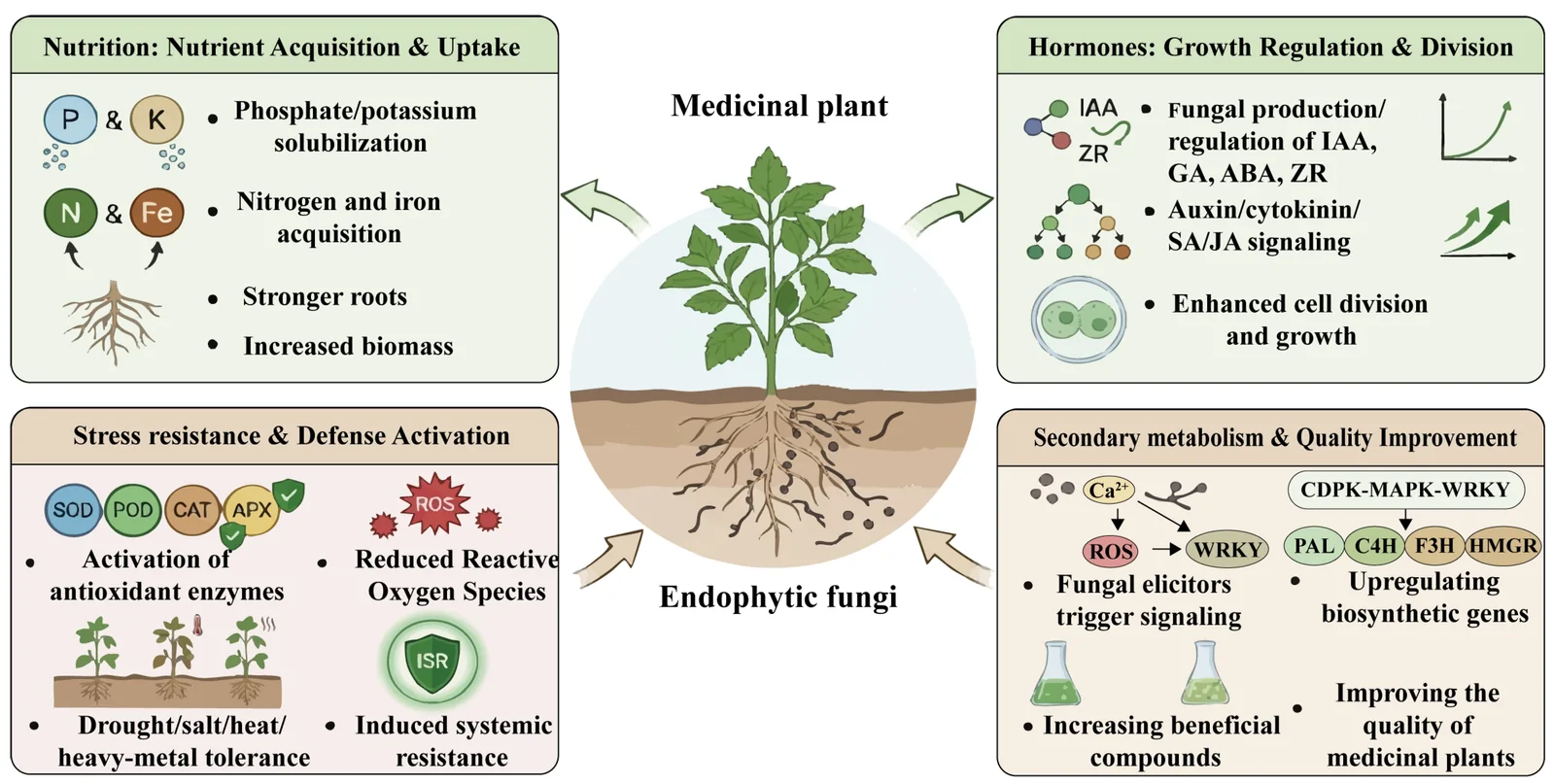
The Promotive Relationship Between Endophytic Fungi and Medicinal Plants: Schematic of four main beneficial effects exerted by endophytic fungi on host medicinal plants. The four modules are interconnected and may operate concurrently through reciprocal interactions and feedback regulation.

**Figure 2:**
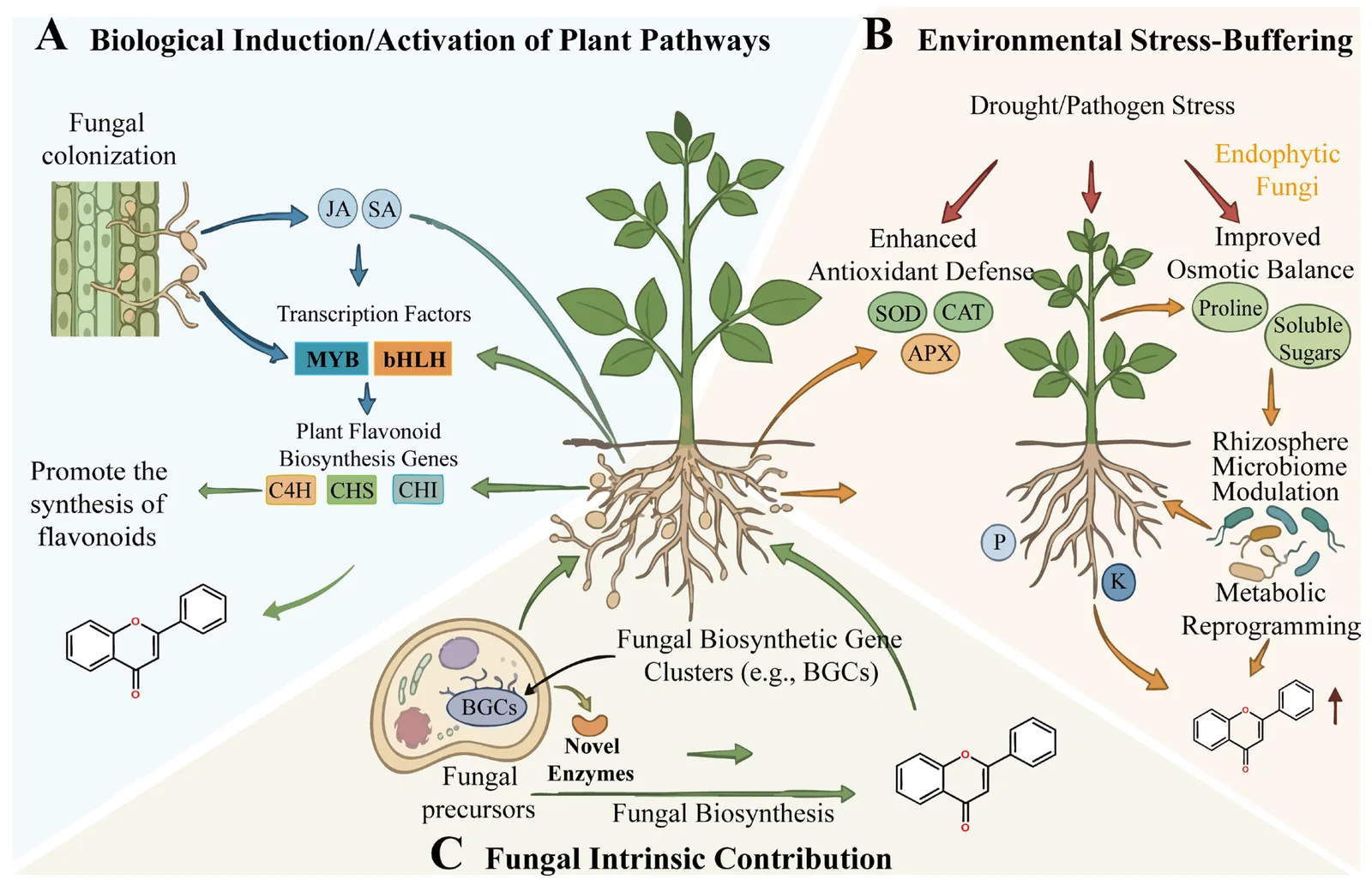
Three regulatory modes of endophytic fungi on flavonoid biosynthesis in medicinal plants. This includes three operational modes: (**A**) biological induction/activation of plant pathways, (**B**) environmental stress-buffering, and (**C**) fungal intrinsic contribution. The overlapping regions indicate that these processes may occur simultaneously and may share signaling, hormonal, and metabolic components. Fungal metabolites may function both as fungal biosynthetic products and as elicitors of host flavonoid metabolism. Stress buffering may influence hormone signaling, reactive oxygen species, carbon allocation, and metabolic flux. Feedback arrows indicate possible reciprocal effects of host flavonoids on fungal recruitment and colonization.

**Figure 3:**
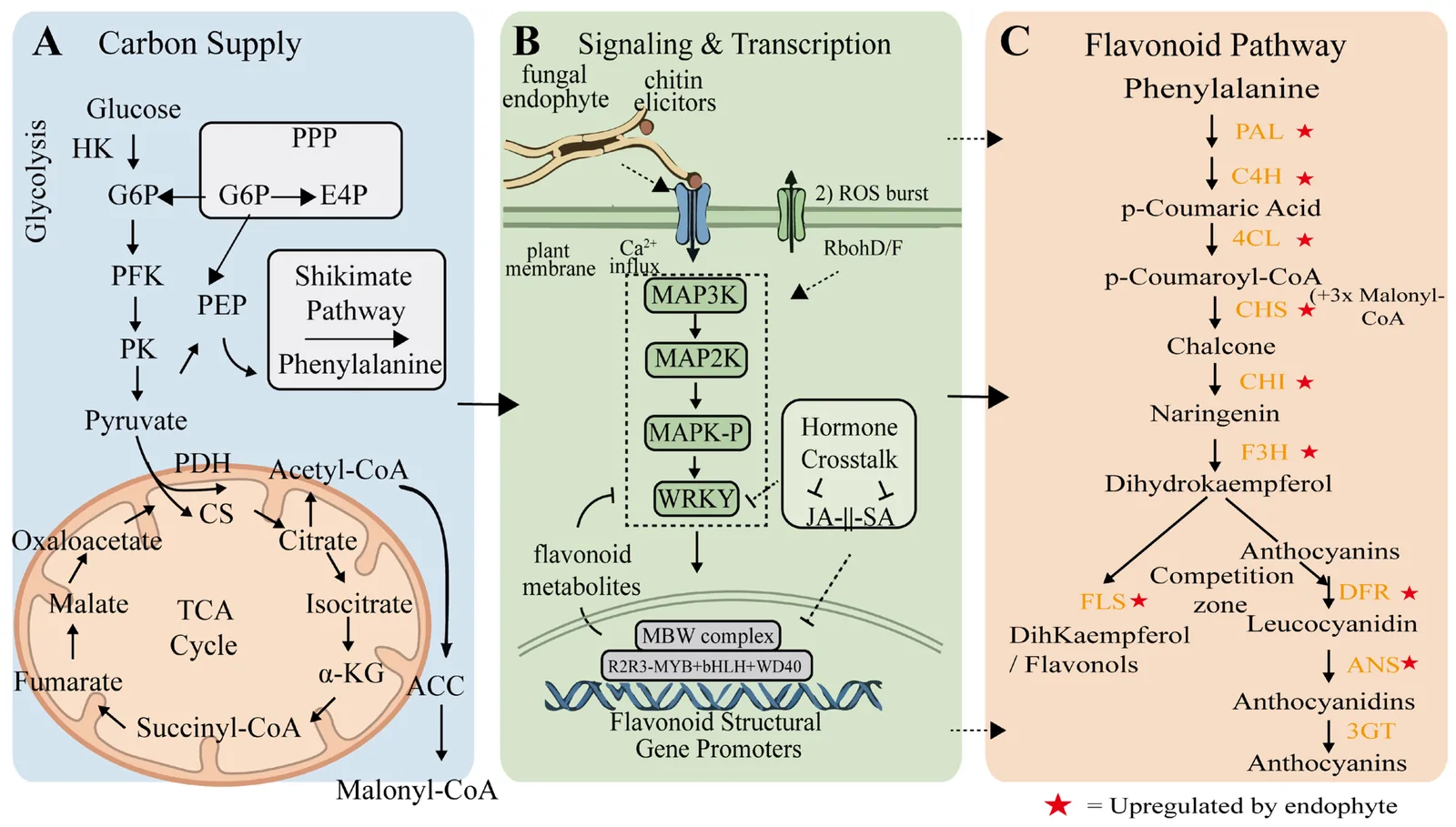
Basic Pathway of Endophytic Fungi Regulating Flavonoid Synthesis Through Biological Induction: (**A**) Primary metabolic pathways supplying carbon precursors (phenylalanine, malonyl-CoA) via glycolysis, PPP, shikimate pathway, and TCA cycle. (**B**) Elicitor-triggered signaling cascade: chitin perception induces Ca^2+^ influx, ROS production, and MAPK/WRKY signaling, converging on the MBW complex to activate flavonoid gene expression, with JA/SA hormone crosstalk involved. (**C**) The flavonoid biosynthetic pathway, where red stars denote endophyte-upregulated key enzymes, including PAL, C4H, 4CL, CHS, CHI, F3H, FLS, DFR, and ANS, leading to flavonol and anthocyanin production via a competition branch point. Solid lines indicate regulatory relationships supported by direct experimental evidence in endophyte–medicinal plant systems; dashed lines represent connections inferred from indirect evidence. Some components of this signaling cascade are canonical plant defense and flavonoid pathways established from general plant molecular research.

**Figure 4:**
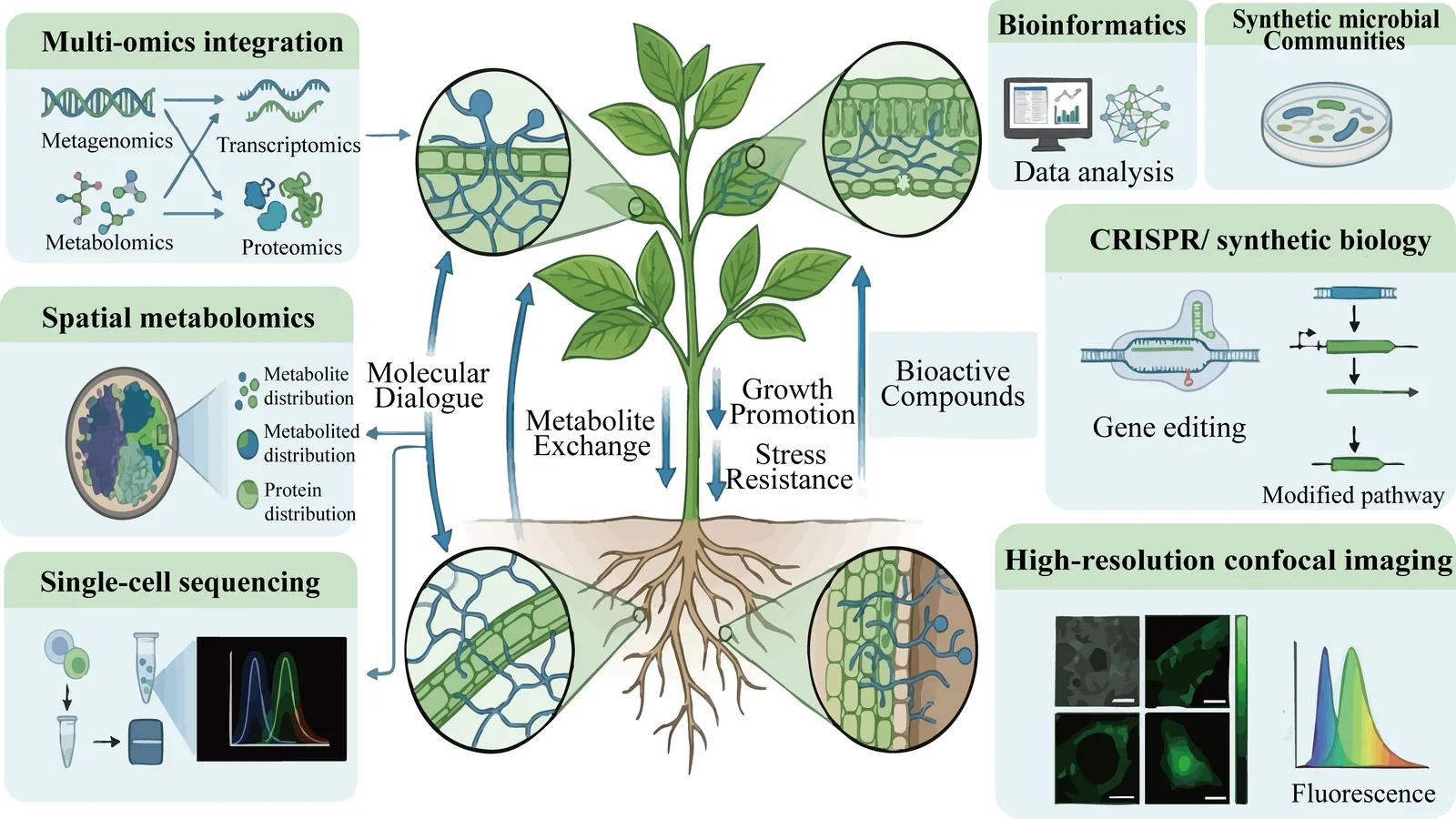
Techniques for studying the interactions between endophytic fungi and medicinal plants. Schematic diagram illustrating multiple advanced technologies applied to decode molecular interactions between medicinal plants and endophytic fungi.

**Table 1:** The Promoting Effects of Endophytic Fungi on Host Medicinal Plants.

Endophytic Fungi	Host Medicinal Plants	Promotional Effects	Key Mechanistic Insight	References
*Plectosphaerella cucumerina*	*Rumex gmelinii Turcz*	Promotes plant growth and secondary metabolism	Upregulates amino acid/carbohydrate metabolism genes	[54]
*Serendipita indica*	*Dendrobium catenatum*	Promotes seed germination and protocorm/corm seedling formation	Enhances early germination and seedling establishment	[55]
*Bipolaris* sp. CSL-1	*Cannabis sativa*	Induces host phenotypic and functional modification	Modulates host physiological and functional traits	[56]
endophytic Cross-Kingdom synthetic microbiota	*Atractylodes chinensis*	Promotes plant growth, secondary metabolism, and fresh weight accumulation	Enhances biomass and secondary metabolic activity	[57]
*Phomopsis* sp. KLBMPSM237	*Salvia miltiorrhiza*	Polysaccharide elicitors enhance phenolic compounds and root biomass	Polysaccharide elicitors stimulate phenolic biosynthesis	[58]
*Penicillium* sp. HJT-A-6	*Rhodiola rosea*	Promotes seed germination and prolongs the germination period	Improves germination performance and duration	[59]
*Fusarium oxysporum* GG22	*Rehmannia glutinosa*	Enhances plant height, leaf area, fresh weight, dry weight, and overall growth	Increases vegetative growth and biomass accumulation	[60]
*Serendipita indica*	*Salix suchowensis*	Enhances stem–leaf biomass and Cd accumulation	Promotes biomass formation and Cd uptake	[61]
*Epichloë guerinii*	*Melica transsilvanica*	Enhances root secretion of organic acids, amino acids, and sugar alcohols	Alters root exudate profile	[62]
*Paraburkholderia*	*Coptis chinensis*	Promotes growth, inhibits root-rot pathogens, and activates immune responses	Activates immunity and suppresses root-rot pathogens	[63]
*Myrmecridium schulzeri* B-4	*Bletilla striata*	Enhances plant growth via IAA synthesis and IAA/BR signaling activation	Produces IAA and activates IAA/BR signaling	[64]
*Trichoderma viride*	*Ashwagandha*	Elevates leaf withanolide A and chlorophyll a contents	Enhances chlorophyll and withanolide A accumulation	[65]
*Glomus versiforme*	*Codonopsis pilosula*	Significantly elevates codonine and atractylolide III contents	Increases bioactive metabolite accumulation	[66]
*Acremonium* sp. Ld-03	*Lilium davidii*	Enhances disease resistance and boosts plant growth	Induces defense responses and growth promotion	[67]
*Piriformospora indica*	*Coleus forskohlii*	Enhances growth, flowering, phosphorus uptake, chlorophyll synthesis, and inflorescence essential-oil accumulation	Improves P uptake, chlorophyll synthesis, and oil yield	[68]

**Table 2:** Endophytic fungi influence the flavonoid biosynthesis pathways in host plants.

Regulatory Mechanism	Mode of Action/Molecular Targets	Host Plant	Endophytic Fungi	Key Results	Evidence Type	References
Contribution of direct metabolites	Fungi synthesize flavonoids themselves	*Ginkgo biloba*.	*Aspergillus* sp. Gbtc 2	Complete flavonoid biosynthesis pathways for independent flavonoid synthesis and host-like metabolite production	Direct in vitro fungal autonomous biosynthesis	[103]
	Synthesize host-like bioactive metabolites	*Fagopyrum tataricum*	*Alternaria* sp.	Enhanced PAL activity, rutin, and total flavonoid levels by fungal polysaccharides	Biochemical + Metabolomic evidence	[104]
	Shared metabolites of fungi and plants	Various Medicinal Plants	*Alternaria*, *Didymella sinensis*	Secreted berberine, catechins, and flavonoids supplement the host flavonoid pool	Metabolomic evidence (in vitro fungal metabolite detection; in-plant contribution not confirmed)	[105,106]
Elicitor effect	Polysaccharide-induced benzyl-propane pathway	*Fagopyrum tataricum*	*Fusarium oxysporum* Fat9	Elevated PAL activity and rutin accumulation by EPS and APS	Biochemical + Metabolomic evidence	[107]
	Extracellular polysaccharide (EPS)-induced	*Scutellaria baicalensis*	*Fusarium solani* CL105	EPS increased baicalin content by 1.40-fold, wogonoside by 1.91-fold, and wogonin by 2.76-fold	Metabolomic + Biochemical evidence	[108]
	Induced by fungal hyphae extract	*Echinacea purpurea*	*Piriformospora indica*	Induced plant defense and flavonoid accumulation via pathogenic mimicry	Physiological + Metabolomic evidence	[109]
Upregulation of key enzyme genes	Activation via the propanediol pathway	*Scutellaria baicalensis*	*Fusarium solani* CL105	Upregulated expression of CHS, CHI, FNS, and F6H genes	Transcriptomic evidence	[108]
	Activation of the PAL gene	*Anoectochilus roxburghii*	*Ceratobasidium* sp. AR2	Upregulated PAL, CHS, 4CL, and GT expression by mycorrhizal fungi	Transcriptomic evidence	[110]
	Induction of key enzymes in the flavonoid biosynthetic pathway	*Astragalus membranaceus*	*Funneliformis mosseae*	Regulated PAL expression and enhanced calycosin-7-glucoside synthesis by inoculation	Transcriptomic + Metabolomic evidence	[111]
Transcription factor regulation	Activation of MYB family transcription factors	*Scutellaria baicalensis*	*F. solani* CL105	Upregulated MYB3/MYB8/MYB13 to modulate flavonoid biosynthesis genes	Transcriptomic evidence	[108]
	Hormone signaling and transcription factor interactions	*Ficus carica*	*Piriformospora indica*	Activated MYB3 and phenylpropanoid pathway to enhance phenolic synthesis in fig hairy roots	Transcriptomic + Metabolomic evidence	[112]
Interactions between plant hormone signals	GA and ABA pathways	*Scutellaria baicalensis*	*F. solani* CL105	Upregulated GA2ox and CYP707A to coordinately modulate flavonoid accumulation via hormones	Transcriptomic + Metabolomic evidence	[108]
	JA and SA signals	Various Medicinal Plants	Various Endophytic Fungi	Fungal elicitors activate JA/SA signaling to induce defense and flavonoid accumulation	Physiological correlative evidence	[113,114,115]
Responding to environmental stress	Mitigation of heavy metal stress	*Astragalus membranaceus*	*Funneliformis mosseae*	Elevated stem total flavonoids, root calycosin (117.1%) and ononin (59.6%) under Cd stress via inoculation	Physiological + Metabolomic evidence	[111]
	Oxidative stress response	*Ginkgo biloba*	*Aspergillus* sp. Gbtc 2	Flavonoids exert antioxidant roles; fungus-induced ROS activates their defensive biosynthesis	Physiological + Biochemical evidence	[103]
	Promotes nutrient absorption	Various Medicinal Plants	Arbuscular Mycorrhizal Fungi (AMF)	Enhanced N/P uptake supplies flavonoid carbon skeletons; flavonoids signal to facilitate mycorrhizal formation	Correlative physiological evidence	[116,117]

## Data Availability

No new data were created or analyzed in this study. Data sharing is not applicable to this article.
